# Supplementary material for: Impact of two field preservation methods on genotyping success of feces
Source: PeerJ. 2025 Oct 8;13:e20154. doi: 10.7717/peerj.20154 (PMC12514995; doi:10.7717/peerj.20154)
Supplement: Supplemental Information 1 [file peerj-13-20154-s001.pdf]

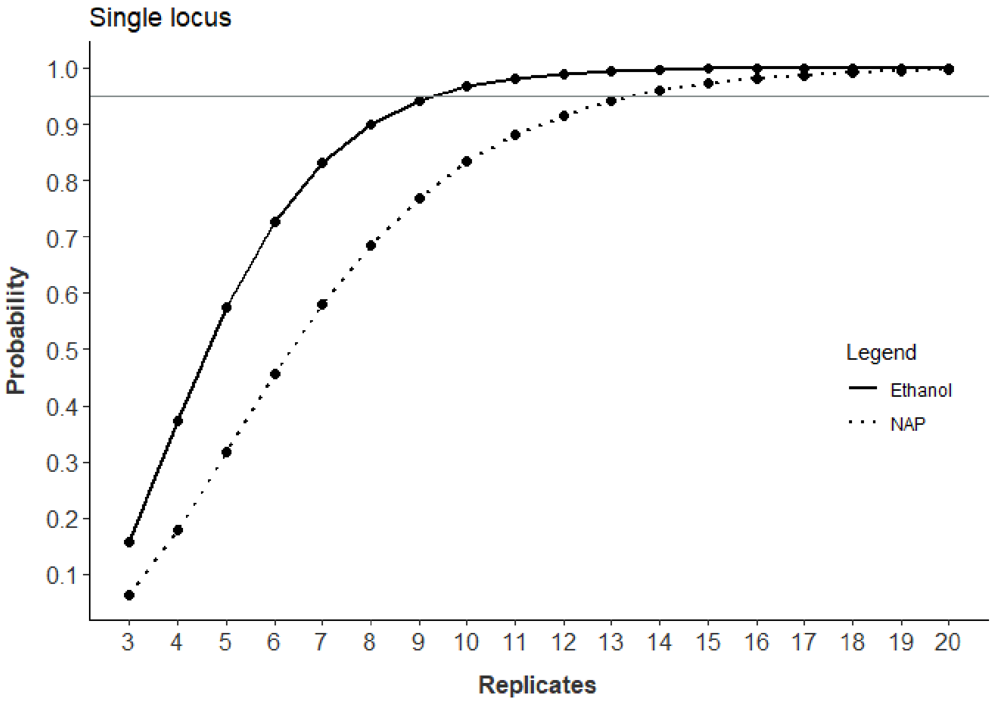

Figure S1. Probability of obtaining the correct genotype at 3 or more replicates for a homozygous locus. using Ethanol and NAP as a preservation method. Calculations are based on the amplification success (AS) and the genotyping success (GS) calculated for each preservation method (**Table 1**). Horizontal line marks probability of 0.95.

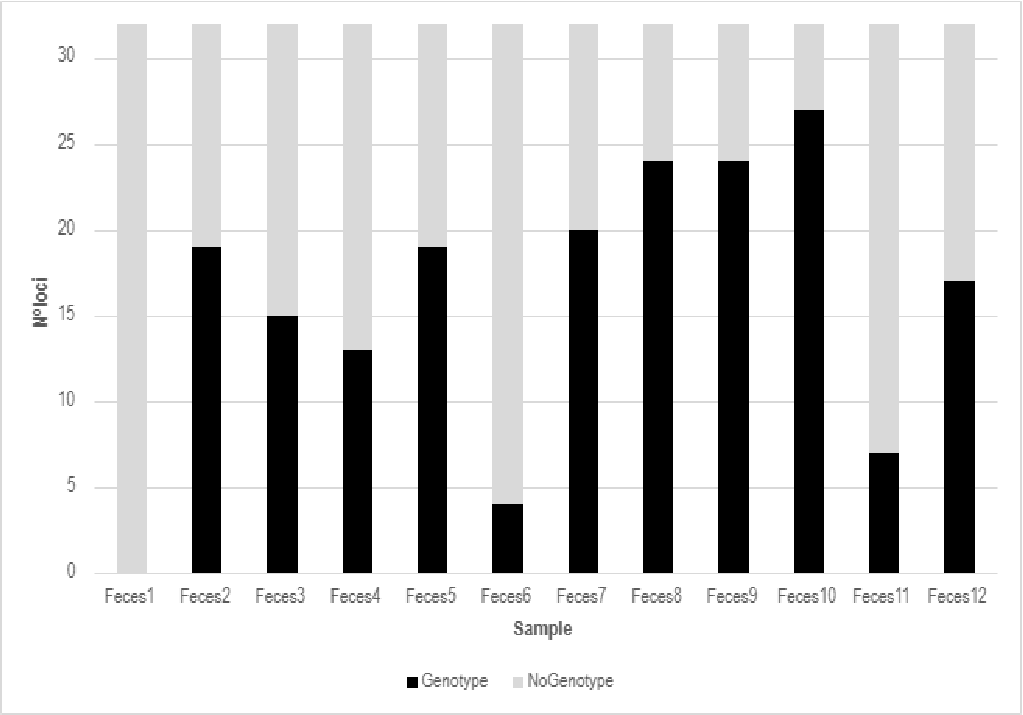

Figure S2. **Completeness of the *reference genotype* obtained for the microsatellite panel for each fecal sample**. Number of loci with a genotype out of the 32 microsatellite loci in the panel for each sample is indicated with the black bar (**Table S4**). Loci without a genotype, marked in grey, were those with no data or considered as ‘ambiguous’ (see Methods).
